# RETRACTION: Prp19 Facilitated p21‐Dependent Senescence of Hepatocellular Carcinoma Cells

**DOI:** 10.1155/jo/9870183

**Published:** 2026-07-01

**Authors:** Journal of Oncology

RETRACTION: J. Yin, G. C. Zhang, Y. Fang, et al., “Prp19 Facilitated p21‐Dependent Senescence of Hepatocellular Carcinoma Cells, *Journal of Oncology*, 2022, 5705896, 9 pages, 2022, https://doi.org/10.1155/2022/5705896.

The above article, published online on 21st March 2022 in Wiley Online Library (https://onlinelibrary.wiley.com), has been retracted by agreement between the journal Editor‐in‐Chief, Jeannette Vasquez‐Vivar; and John Wiley & Sons Ltd. The retraction has been agreed due to concerns regarding some images in the article. Specifically, a third party reported that the western blots representing the Huh7 experiments shown in figure 4(a) are duplicated from an image in Figure 3C published in a different article [1]. In addition, further investigation found that the untreated mock panel in figure 2(a) is a duplication from figure 1(a) in the same article. The authors were asked for the raw data and an explanation, but their explanation was not considered sufficient by the journal. The results are therefore considered unreliable, and the article therefore must be retracted. The authors disagree with the retraction.
